# Exercise Training Prevents Cardiovascular Derangements Induced by Fructose Overload in Developing Rats

**DOI:** 10.1371/journal.pone.0167291

**Published:** 2016-12-08

**Authors:** Daniela Farah, Jonas Nunes, Michelle Sartori, Danielle da Silva Dias, Raquel Sirvente, Maikon B. Silva, Patrícia Fiorino, Mariana Morris, Susana Llesuy, Vera Farah, Maria-Cláudia Irigoyen, Kátia De Angelis

**Affiliations:** 1 Laboratory of Metabolic Cardiovascular and Renal Physiopharmacology, Universidade Presbiteriana Mackenzie, Sao Paulo, Brazil; 2 Laboratory Experimental Hypertension, Heart Institute (INCOR), School of Medicine, Sao Paulo University (FMUSP), Sao Paulo, Brazil; 3 Laboratory of Translational Physiology, Universidade Nove de Julho, Sao Paulo, Brazil; 4 Institute Neuro Immune Medicine, Nova Southeastern University, Fort Lauderdale, United States of America; 5 Department of General and Inorganic Chemistry, University of Buenos Aires, Buenos Aires, Argentina; Max Delbruck Centrum fur Molekulare Medizin Berlin Buch, GERMANY

## Abstract

The risks of chronic diseases associated with the increasing consumption of fructose-laden foods are amplified by the lack of regular physical activity and have become a serious public health issue worldwide. Moreover, childhood eating habits are strongly related to metabolic syndrome in adults. Thus, we aimed to investigate the preventive role of exercise training undertaken concurrently with a high fructose diet on cardiac function, hemodynamics, cardiovascular autonomic modulation and oxidative stress in male rats after weaning. Male Wistar rats were divided into 4 groups (n = 8/group): Sedentary control (SC), Trained control (TC), Sedentary Fructose (SF) and Trained Fructose (TF). Training was performed on a treadmill (8 weeks, 40–60% of maximum exercise test). Evaluations of cardiac function, hemodynamics, cardiovascular autonomic modulation and oxidative stress in plasma and in left ventricle (LV) were performed. Chronic fructose overload induced glucose intolerance and an increase in white adipose tissue (WAT) weight, in myocardial performance index (MPI) (SF:0.42±0.04 vs. SC:0.24±0.05) and in arterial pressure (SF:122±3 vs. SC:113±1 mmHg) associated with increased cardiac and vascular sympathetic modulation. Fructose also induced unfavorable changes in oxidative stress profile (plasmatic protein oxidation- SF:3.30±0.09 vs. SC:1.45±0.08 nmol/mg prot; and LV total antioxidant capacity (TRAP)- SF: 2.5±0.5 vs. SC:12.7±1.7 uM trolox). The TF group showed reduced WAT, glucose intolerance, MPI (0.35±0.04), arterial pressure (118±2mmHg), sympathetic modulation, plasmatic protein oxidation and increased TRAP when compared to SF group. Therefore, our findings indicate that cardiometabolic dysfunctions induced by fructose overload early in life may be prevented by moderate aerobic exercise training.

## Introduction

The world is consuming each year more sugar from high fructose corn syrup (HFCS) containing 42–55% of fructose and commonly found in beverages and industrial foods [1]. The resulting increase in cases of morbidity and mortality such as obesity, hypertension and diabetes [2] have encouraged several studies to focus on the risk factors posed by this trend [3, 4] and the preventive strategies to fight them [5].

High-fructose diet in rats can cause metabolic syndrome, as previously demonstrated by our group [6, 7]. We have shown that a high fructose diet negatively affects the cardiovascular autonomic control on both sexes in adult rats [6, 8]. Experimental models of metabolic syndrome have demonstrated increased oxidative stress by decreasing catalase concentration and increasing lipoperoxidation [9, 10, 11]. Girard et al. [12] have demonstrated that a fructose-enriched diet alters the redox balance in the blood and in the lipid metabolism in spontaneously hypertensive rats.

On the other hand, the benefits of non-pharmacological and non-invasive therapies, such as exercise training (ET), are becoming increasingly acknowledged. In this context, ET may be implemented to modify lifestyle and reduce risk factors associated with metabolic syndrome, since it has been shown to be clinically relevant to fight the negative effects of the Western diet [13, 14]. The ET-associated autonomic nervous system benefits have been largely demonstrated by several studies. ET improves baroreflex control of heart rate (HR) in both sexes and also plays an important role on resting HR [15, 16]. Studies have suggested that these improvements in HR may be due to increased vagal activity [17]. Boa et al. [18] have demonstrated that aerobic ET undertaken for 4 weeks, following 20 weeks of fructose overload in young male hamsters, may improve microvascular and endothelium dysfunction.

However, there have been very few studies focusing on recent weaned rats. In this sense, 9 weeks of fructose-rich diet immediately after weaning display impaired hepatic insulin sensitivity, but did not cause significant changes in the liver antioxidant enzymes or markers of damage induces by oxygen reactive species, suggesting that hepatic changes might be attributed to molecular mechanisms other than redox imbalance in this model [19].

Therefore, the childhood eating habits are strongly related to metabolic syndrome in adults and the risks of chronic diseases associated with the increasing consumption of fructose-laden foods are amplified by the lack of regular physical activity. Considering these, in the present study we aimed to investigate the preventive role of exercise training undertaken concurrently with a high fructose diet (10% on the drinking water) on cardiac function, hemodynamics, cardiovascular autonomic modulation and oxidative stress in male rats after weaning.

## Materials and Methods

### Animals

Experiments were performed in male Wistar rats (21-days-old to 50 ± 10 g). Animals were obtained from the University of Sao Paulo, Sao Paulo, Brazil. Rats received standard laboratory chow and water *ad libitum*. The animals were housed in cages with four each in a temperature-controlled room (22°C) with a 12-h dark-light cycle. The rats were randomly assigned to one of the four groups: sedentary control (SC; *n* = 8), trained control (TC; n = 8) sedentary high-fructose (SF; *n* = 8), and trained high-fructose (TF; *n* = 8). All protocols were in accordance with the Guidelines for Ethical Care of Experimental Animals from the International Animal Care and Use Committee. This study was approved by the Mackenzie University Ethical Committee (CEAU-UPM 053/08/2009) and by Universidade Nove de Julho Ethical committee (CEAU-UNINOVE 003/2012).

### High-fructose diet

The SF and TF groups received an overload of 10% of D-fructose (LabSynth, Sao Paulo, Brazil) in the drinking water for 8 weeks after weaning (21 days old). Control animals received only water [8].

### Exercise training

Moderate-intensity exercise training (40–60% maximal running speed, 0% inclination) was performed on a treadmill once a day at the same time every day, 5 days/week during 8 weeks, as described in detail elsewhere [5]. The protocol was initially carried out with the concurrent administration of fructose.

All animals were adapted to the procedure (10 min/day; 0.3 km/h) for 1 week before the beginning of the exercise training protocol. Sedentary and trained groups underwent a maximal treadmill test [20]. The tests were performed in weeks 1, 4, and 8 of exercise training to determine aerobic capacity and adequate exercise training intensity.

### Metabolic evaluations

Chow and water (with or without fructose) consumption were measured weekly. The total caloric intake was calculated using 2.89 kcal per gram of chow consumed and that each ingested gram of fructose corresponds to 4.0 kcal.

After 8 week of fructose overload, glucose tolerance test of all animals was performed after a 12-h fasting with a specific device (Accu-Check Advantage Blood Glucose Monitor, Roche Diagnostic Corporation, Indianapolis. IN). Animals received a dose of 1.5 g/kg body weight intraperitoneally (intraperitoneal glucose) and a drop of blood from the end of the tail was collected at baseline and 15, 30, 60, 90 and 120 minutes after the glucose injection [21]. Triglycerides and total cholesterol were analyzed in the serum by the chemiluminescent method (Lab test, Brazil).

### Echocardiographic evaluation

In the final week of training, echocardiography was performed according to the guidelines of the American Society of Echocardiography. The rats were anesthetized with ketamine 80 mg/kg + xylazine 12 mg/kg IP, and images were obtained with the transducer on each animal's shaved chest (lateral recumbence) using a 10–14 MHz linear transducer in a SEQUOIA 512 (ACUSON, Mountain View, CA, USA) to measure morphometric parameters such as left ventricular mass (LVM), LV cavity in diastole correct by weight (LVd weight), and relative wall thickness (RWT); systolic function was evaluated by ejection fraction (LVEF), fractional shortening (FEFS); while diastolic function was evaluated by the relation between maximal early diastolic peak velocity and late peak velocity (E/A), deceleration of early diastolic peak velocity, and isovolumetric relaxation time (IVRT). A global index was quantified by the myocardial performance index (MPI). All measurements were based on the average of 3 consecutive cardiac cycles. Echocardiography parameters were measured as previously described [21, 22].

### Cardiovascular assessments

After 24 hours of the last training session, a catheter filled with 0.06 mL of saline was implanted into the femoral artery while the animals were anesthetized (Ketamine 80 mg/kg + Xylazine 12 mg/kg) for direct measurements of arterial pressure (AP).

Rats were studied 24 hours after catheter placement. The animals were conscious and allowed to move freely during the experiments. An arterial cannula was connected to a strain-gauge transducer (Blood Pressure XDCR, Kent Scientific, Litchfield, CT, USA), and AP signals were recorded over a 30-min period by a microcomputer equipped with an analog-to-digital converter board (Windaq, 2-kHz sampling frequency; Dataq Instruments, Akron, OH). The recorded data were analyzed on a beat-to-beat basis to quantify changes in mean AP and heart rate (HR). HR variability (HRV) was determined by using the standard deviation of the basal HR recording period [5].

### Arterial pressure and pulse interval variability evaluation

The overall variability of pulse interval (PI) and systolic arterial pressure (SAP) was assessed in the time domain by means of variance. PI and SAP fluctuations were assessed in the frequency domain using autoregressive spectral analysis, as described elsewhere [23, 24]. Briefly, PI and SAP series were divided in segments of 300 beats and overlapped by 50%; a spectrum was obtained for each of the segments via the Levinson-Durbin recursion, with the model order chosen according to Akaike's criterion, ranging between 10 and 14. The oscillatory components were quantified in the low- (LF; 0.1 to 1 Hz) and high-frequency ranges (HF; 1 to 5.0 Hz). The LF band for SAP and PI oscillations was similar to that used by Janssen et al. [25]. However, unlike these authors, we set the upper limit for the HF band at 5 Hz, since no spectral component was found beyond this frequency. The power spectrum density was calculated for each recognizable component in the LF and HF bands by integrating the spectrum of the components. Segments that presented very slow oscillations (<0.1 Hz), which contributed to >70% of the overall variability, were considered nonstationary and discarded from the study. The sympathovagal balance was defined by the LF normalized unit (nu) to HF nu ratio. The alpha index analysis evaluates short-term changes in the systolic blood pressure and in the RR interval. This method has been proposed to quantify cause-and-effect events linked with the baroreflex [5].

### Oxidative stress evaluation

After all metabolic and cardiovascular measurements were taken animals were pre anesthetized (Ketamine 80 mg/kg + Xylazine 12 mg/kg) and were euthanized by decapitation. The LV was immediately removed, rinsed in saline, and trimmed to remove fat tissue and visible connective tissue, and also, plasma was collected. The LV was cut into small pieces, placed in ice-cold buffer, and homogenized in an ultra-Turrax blender with 1 g of tissue per 5 mL of 150 mmol/L KCl and 20 nmol/L phosphate buffer, pH 7.4. The homogenates were centrifuged at 600g for 10 minutes at −2°C. Proteins were assayed by the method of Lowry *et al* [26].

Lipoperoxidation was evaluated by TBARS assay and by chemiluminescence initiated by tert-butil (CL). For TBARS the trichloroacetic acid (10%. w/v) was added to the LV homogenates to precipitate proteins and to acidify the samples (LV and plasma) [27]. This mixture was then centrifuged (1000G. 3 minutes), the protein-free sample was extracted, and thiobarbituric acid (0.67%. w/v) was added to the reaction medium. The tubes were placed in a water bath (100°C) for 15 minutes. Absorbance was measured at 535 nm using a spectrophotometer. The CL assay was performed in LV and carried out with an LKB Rack Beta liquid scintillation spectrometer 1215 (LKB Producer AB), in the out-of-coincidence mode at room temperature (25°C to 27°C). Supernatants were diluted in 140mmol/L KCl and 20mmol/L phosphate buffer, pH 7.4, and added to glass tubes, which were placed in scintillation vials; 3mmol/L tert-butylhydroperoxide was added, and CL was determined up to the maximal level of emission [9,28].

Protein oxidation uses the reaction of protein carbonyl groups with 2.4- *dinitro phenylhydrazine* (DNPH) to form a 2.4-dinitrophenylhydrazone. The product of the reaction was measured at 360 nm, as previously described [29]. The concentration of the carbonyl in the cardiac tissue and plasma was standardized by protein concentration (nmol carbonyl group/mg protein). The amount of protein oxidation was calculated from bovine serum albumin dissolved in guanidine hydrochloride and read at 280 nm.

Catalase (CAT) activity in the heart was measured spectrophotometrically by monitoring the decrease in H_2_O_2_ concentration over time. Aliquots of the samples were added to 50 mmol/L phosphate buffer in a quartz cuvette. After determining the baseline of the instrument, H_2_O_2_ was added to a final concentration of 10 mmol/L in 0.9 mL, and absorbance was measured at 240 nm [30]. Superoxide dismutase (SOD) activity was measured in the heart and plasma by measuring the inhibition of the rate of autocatalytic adrenochrome formation at 480 nm in a reaction medium containing 1 mmol/L epinephrine and 50 mmol/L glycine-NaOH, pH 10.5 [31]. Glutathione peroxidase (GPx) activity was assessed at the heart by adding to the assay a mixture of 1 U/mL glutathione reductase and 2 mmol/L glutathione in 1 mL phosphate buffer. Mixtures were preincubated at 37°C for 30 minutes. Subsequently, NADPH and *tert*-butylhydroperoxide were added, and the change in absorbance at 340 nm was recorded to calculate GPx activity, as previously described [32].

The level of total antioxidant capacity (TRAP) was determined by measuring luminol chemiluminescence intensity induced by the thermolysis of 2.2'-azobis(2-amidinopropane) dihydrochloride in plasma and LV. The results are expressed as μM of 6-hydroxy-2.5.7.8-tetramethylchroman-2-carboxylic acid per mg of protein [33].

For evaluation of glutathione redox balance (GSH/GSSG) the LV was deproteinized with 2 mol/L perchloric acid and centrifuged for 10 minutes at 1,000g, and the supernatant was neutralized with 2 mol/L potassium hydroxide. The reaction medium to determination of reduced glutathione (GSH) and oxidized glutathione (GSSG) concentrations contained 100mmol/L phosphate buffer (pH7.2), 2mmol/L nicotinamidedinucleotide phosphate acid, 0.2 U/mL glutathione reductase, and 70 mmol/L 5,50dithiobis (2-nitrobenzoicacid). For determination of GSSG concentration, the supernatant was neutralized with 2 mol/L potassium hydroxide for reaction with 70 mmol/L 5,50 dithiobis (2-nitrobenzoic acid), and absorbance values were measured at 420 nm [9,28].

### Statistical analysis

Data were analyzed post-intervention and are reported as means ± SE. Levene’s test was used to assess variance homogeneity. ANOVA (two-way) was used to compare groups, followed by the Student-Newman-Keuls post hoc test when applied. Significance was considered when *p* < 0.05.

## Results

### Metabolic evaluations

The group SF was significant higher in glucose tolerance as analyzed by area under curve when compared to all groups, and the group TC was lower when compared to all groups. The plasmatic levels of total cholesterol was increased in SF group in relation to SC and TC groups (SF: 138 ± 15 vs. SC: 90 ± 11 TC: 72 ± 7 mg/dl). The TF group (113 ± 16 mg/dl) presented similar values of total cholesterol than sedentary groups. The plasmatic levels of triglycerides were increased in SF group among all studied groups (SF: 151 ± 7 vs. SC: 74 ± 4, TC: 52 ± 4, TF: 126 ± 8 mg/dl). The trained groups showed reduced triglycerides when compared to sedentary groups.

**Fig 1:**
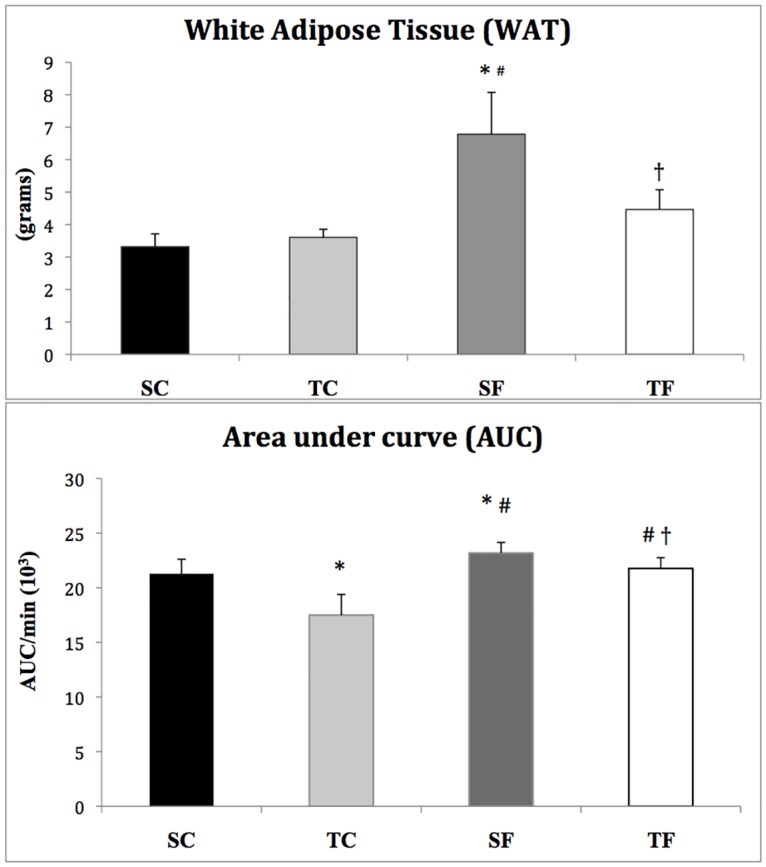
Abdominal fat tissue and glucose intolerance (AUC) in sedentary control (SC), trained control (TC), sedentary fructose (SF) and trained fructose (TF) rats. * p<0.05 vs SC; # p<0.05 vs TC; † p<0.05 vs SF.

The body weight gain was similar between groups (SC: 259 ± 8, TC: 263 ± 8, SF: 256 ± 10, TF: 253 ± 7 g). However, the SF group showed increased abdominal fat tissue when compared to others (Fig 1). Chow consumption was higher in control groups (SC: 28 ± 0.6 and TC: 28 ± 0.1 g·day-1·rat-1) in relation to fructose-fed groups (SF: 19 ± 1.7 and TF: 20 ± 0.8 g·day-1·rat-1). The fructose-fed animals, however, presented statistically significant greater water consumption (SF: 65 ± 5.4 and TF: 60 ± 0.9 ml·day-1·rat-1) compared to control rats (SC: 44 ± 2.4 and TC: 50 ± 1.6 ml·day-1·rat-1). The total caloric intake (chow + fructose) was similar between groups SC: 81 ±1.8; TC: 81 ± 0.3; SF: 82 ± 4.2; TF: 81 ± 2.5 kcal/day).

### Physical performance

The trained groups showed better performance when compared to sedentary at the end of protocol (TC: 2.15±0.05; TF: 2.18±0.07 vs. SC: 1.5±0.05; SF: 1.89±0.07 Km/h). SF showed statistically better performance than SC.

### Echocardiography evaluations

No differences were found in morphological parameters, such as LVM correct by weight, LVDIA correct by weight and RWT between groups. Likewise, no differences were found among groups in both systolic and diastolic function in all parameters measured (Table 1).

**Table 1:** Echocardiographic evaluations in sedentary control (SC), trained control (TC), sedentary fructose (SF) and trained fructose (TF) groups.

	SC	TC	SF	TF
**LVM corr** (mg/g)	3.62 ± 0.12	3.74 ± 0.13	3.59 ± 0.08	3.42 ± 0.06
**LVd corr** (mm/g)	2.10 ± 0.13	2.02 ± 0.07	2.26 ± 0.07	2.01 ± 0.06
**RWT**	0.45 ± 0.033	0.50 ± 0.014	0.44 ± 0.037	0.51 ± 0.038
**LVEF** (%)	81 ± 2.2	84 ± 1.0	77 ± 1.8	84 ± 2.3
**LVFS** (%)	47 ± 2	48 ± 1	41 ± 2	48 ± 3
**IVRT** (ms)	23.3 ± 1.7	23.4 ± 0.7	25.2 ± 2.0	24.7 ±1.0
**E/A**	1.63 ± 0.07	1.71 ± 0.18	1.63 ± 0.10	1.90 ± 0.10
**Desac. E** (ms)	39.3 ± 1.4	40.17 ± 0.8	38.0 ± 2.9	39.2 ± 1.3

Data are expressed as mean ± SEM. LVM corr = left ventricular mass corrected by body weight; LVd /corr = left ventricle cavity in diastole correct by weight; RWT = relative wall thickness; LVEF = left ventricle ejection fraction; LVFS = left ventricle fractional shortening; E/A = relation of maximal early diastolic peak velocity and late peak velocity; Desac. E = desaceleration of early diastolic peak velocity; IVRT = isovolumetric relaxation time.

* p<0.05 vs SC

^#^ p<0.05 vs TC

^†^ p<0.05 vs SF

**Fig 2:**
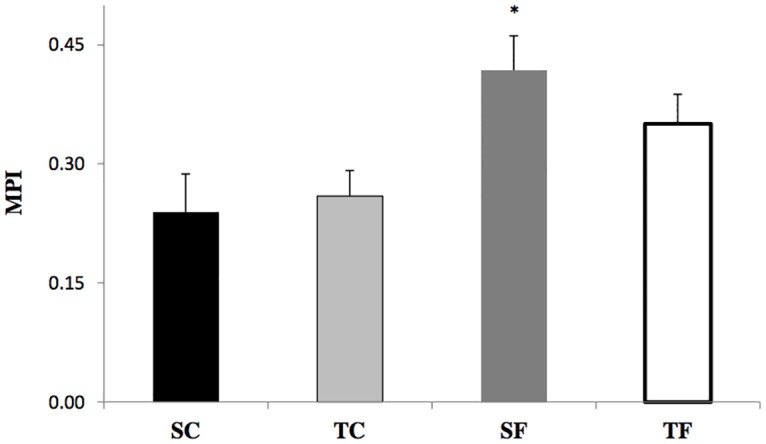
Cardiac global function was evaluated by the myocardial performance index (MPI) in sedentary control (SC), trained control (TC), sedentary fructose (SF) and trained fructose (TF) rats. * p<0.05 vs SC.

MPI, an index of cardiac overload, was increased in SF group when compared to SC and TC groups (SC: 0.24±0.05; TC: 0.26±0.03; SF: 0.42±0.04; TF: 0.35±0.04) (Fig 2).

### Hemodynamic and autonomic modulation

When compared to SC group, the SF group showed a significant increase in MAP, along with increased DAP. The TF group showed decreased DAP when compared to SF group, but no change in MAP was found. The trained groups presented resting bradycardia when compared to sedentary groups (Table 2).

**Table 2:** Hemodynamic and autonomic modulation in sedentary control (SC), trained control (TC), sedentary fructose (SF) and trained fructose (TF) groups.

	SC	TC	SF	TF
**SAP** (mmHg)	138 ± 2	135 ± 4	146 ± 3	140 ± 2
**DAP** (mmHg)	95 ± 2	99 ± 2	106 ± 3 *	103 ± 2*
**MAP** (mmHg)	113 ± 1	115 ± 3	122 ± 3*	118 ± 2
**HR** (bpm)	366 ± 13.4	339 ± 5.8*	378 ± 8.3 ^#^	340 ± 6.7 *^†^
**PIV**				
**SD** (ms)	6.27 ± 0.78	6.66 ± 0.85	7.23 ± 0.59	7.32 ± 0.53
**VAR** (ms^2^)	33.8 ± 4.60	43.0 ± 8.20	50.3 ± 7.51	60.5 ± 5.81
**LF** (ms^2^)	1.32 ± 0.35	1.77 ± 0.45	3.97 ± 0.76*^#^	2.03 ± 0.17^†^
**HF** (ms^2^)	6.32 ± 1.48	9.52 ± 2.12	7.71 ± 1.24	6.82 ± 0.84
**%LF** (nu)	21.65 ± 3.54	23.34 ± 0.93	33.29 ± 2.89*^#^	25.32 ± 2.03^†^
**%HF** (nu)	78.35 ± 3.54	76.66 ± 0.93	66.71 ± 2.89*^#^	74.68 ± 2.03^†^
**SAPV**				
**SD** (mmHg)	4.77 ± 0.20	4.80 ± 0.35	6.16 ± 0.40 *^#^	4.65 ± 0.18 ^†^
**VAR** (mmHg^2^)	25.42 ± 2.77	23.05 ± 3.76	41.75 ± 4.88 *^#^	23.58 ± 2.19 ^†^
**LF** (mmHg^2^)	6.94 ± 0.82	3.72 ± 0.28	11.91 ± 1.08 *^#^	4.80 ± 0.38 ^†^

Data are reported as mean ± SEM. MAP = mean arterial pressure; SAP = systolic arterial pressure; DAP = diastolic arterial pressure; HR = heart rate; PIV = pulse interval variability; SD = standard deviation; VAR = variability; LF = low frequency band; HF = high frequency band; %LF = normalized low frequency band; %HF = normalized high frequency band; SAPV = systolic arterial pressure variability.

* p<0.05 vs SC

^#^ p<0.05 vs TC

^†^ p<0.05 vs SF

**Fig 3:**
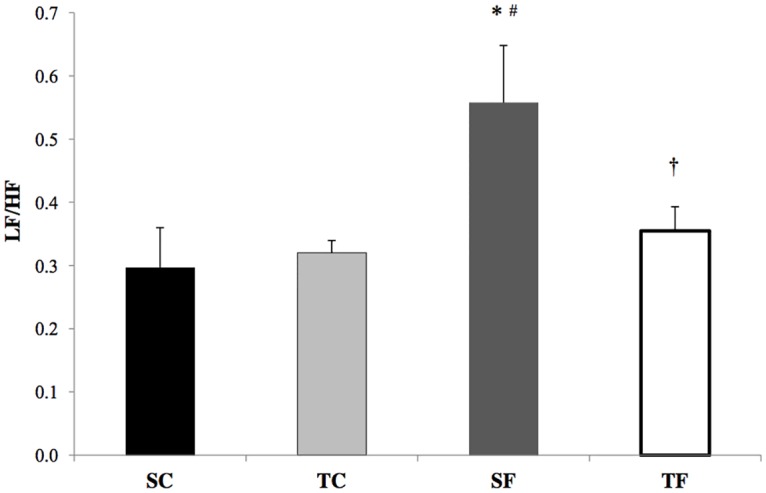
Sympathovagal balance (LF/HF) in sedentary control (SC), trained control (TC), sedentary fructose (SF) and trained fructose (TF) groups. * p<0.05 vs SC; # p<0.05 vs TC; † p<0.05 vs SF.

Data regarding HRV and SAPV in the time and frequency domains are shown in Table 2. Standard deviation of PI (SD-PI), PI variance (PI-var), and HF did not show significant differences among groups. The LF of PI showed a significant increase in SF when compared to the other groups. Sympathovagal balance was greater on SF when compared to the other groups (Fig 3).

The standard deviation of SAP (SD-SAP), SAP variability (SAP-var) and LF-SAP were significant greater in SF group.

The alpha index, which indicates spontaneously barorreflex sensitivity, was not different among the groups (SC: 0.53 ± 0.06; TC: 0.77 ± 0.14; SF: 0.57 ± 0.07; TF: 0.73 ± 0.05 ms/mmHg).

### Oxidative stress

**Fig 4:**
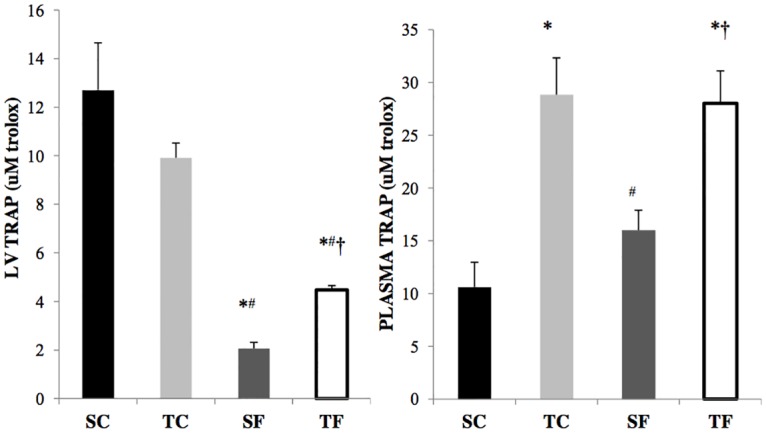
Total antioxidant capacity (TRAP) on left ventricle (LV) and plasma in sedentary control (SC), trained control (TC), sedentary fructose (SF) and trained fructose (TF) groups. * p<0.05 vs SC; # p<0.05 vs TC; † p<0.05 vs SF.

Oxidative stress parameters are shown in Table 3. Lipoperoxidation in LV, as evaluated by TBARS, was decreased in TF group when compared to all groups. The CL evaluation demonstrated increased lipoperoxidation in LV in SF group as compared to other studied groups. Protein oxidation was also reduced in TF rats when compared to SC. Regarding antioxidant enzymes in LV, CAT was increased in TC group in relation to SC group. A decrease in SOD was observed in SF group when compared to SC group. The GPx in LV was not different between the groups (Table 3). The evaluation of TRAP in LV was decreased in the groups consuming fructose when compared to controls (SC: 12.70 ± 1.70; TC: 8.83 ± 2.62 vs. SF: 2.06 ± 0.52; TF: 4.48 ± 0.20 μM trolox). The TF group presented increased TRAP in when compared to SF group (Fig 4). The glutathione redox balance, evaluated by GSH/GSSG, was increased in TF group in relation to other studied groups (Table 3).

**Table 3:** Oxidative stress assessment in left ventricle (LV) and plasma in sedentary control (SC), trained control (TC), sedentary fructose (SF) and trained fructose (TF) groups.

	SC	TC	SF	TF
**LV**				
**Lipoperoxidation**				
TBARS (μmol/mg protein)	5.36 ± 0.40	4.85 ± 0.82	4.33 ± 0.35	2.95 ± 0.20 *^#^^†^
CL (cps/mg protein)	5846 ± 215	5795 ± 186	6800 ± 296*^#^	5255 ± 212 ^†^
Protein Oxidation (nmol/mg protein)	4.71 ± 0.51	3.85 ± 0.45	3.22 ± 0.34	2.89 ± 0.37 *
CAT (nmol/mg protein)	0.26 ± 0.02	0.42 ± 0.03*	0.31 ± 0.03	0.33 ± 0.03
SOD (USOD/mg protein)	14.91 ± 0.54	14.26 ± 0.88	11.98 ± 0.75*	12.61 ± 0.94
GPx (μmol/min/mg protein)	0.029 ± 0.001	0.035 ± 0.008	0.029 ± 0.002	0.035 ± 0.001
GSH/GSSG	11.95 ± 0.52	11.25 ± 0.66	12.94 ± 0.53	16.17 ± 0.33 *^#^^†^
**PLASMA**				
**Lipoperoxidation**				
TBARS (μmol/mg protein)	0.10 ± 0.01	0.10 ± 0.01	0.19 ± 0.03 *^#^	0.19 ± 0.02 *^#^
Protein Oxidation (nmol/mg protein)	1.45 ± 0.08	1.49 ± 0.11	3.30 ± 0.09 *^#^	2.75 ± 0.18 *^#^^†^
SOD (USOD/mg protein)	1.00 ± 0.03	0.98 ± 0.05	0.95 ± 0.03	0.91 ± 0.04

Data are reported as mean ± SEM. Antioxidant enzymes: SOD = Superoxide dismutase; GPx = Glutathione peroxidase; CAT = Catalase.

* p<0.05 vs SC

^#^ p<0.05 vs TC

^†^ p<0.05 vs SF

The evaluations of oxidative stress on plasma showed an increase in lipoperoxidation and protein oxidation in the fructose groups when compared to groups consuming water. No significant changes in plasmatic SOD were found between the groups (Table 3).

TRAP on plasma showed an increase in trained groups when compared to the sedentary ones (TC: 28.8 ± 3.49; TF: 28.04 ± 3.05 vs SC: 10.60 ± 2.34; SF: 15.98 ± 1.98 μM trolox) (Fig 4).

## Discussion

Our results showed metabolic, hemodynamic and autonomic dysfunction accompanied by oxidative stress derangements in animals treated with fructose and an attenuation of these changes in trained animals.

The metabolic alterations observed in our results are in accordance with others studies from our group. The fructose overload induced glucose intolerance and/or insulin resistance, unfavorable changes in lipid profile, as well as an increase in adipose tissue in male and female adults and developing animals [6, 7, 8, 21]. Indeed, our study showed that physical training was able to prevent these metabolic alterations induced by fructose overload as previous reported by others studies [5, 17, 34, 35, 36].

Regarding physical capacity evaluation, the trained animals showed improvement in physical capacity when compared to sedentary groups. In the literature, the improvement in physical function is viewed as a marker of physical training protocol efficiency; this improvement has been widely reported after training in control, diabetic, old and hypertensive rats [17, 35, 37, 38, 39, 40] as well as in healthy men and hypertensive and in post-myocardial infarction patients [41, 42, 43]. Our group also demonstrated cardiovascular autonomic improvement after aerobic exercise training in adult male and ovariectomized rats undergoing a fructose overload [34, 36]. Additionally, we observed an increase in the maximum exercise test in hypertensive ovariectomized rats undergoing a fructose consumption and aerobic exercise training [35]. Interestingly, the SF group reached a higher speed in the maximum exercise test than the SC group. This finding could be linked to the sympathetic activation induced by chronic consumption of fructose, as observed in this study. However, it should be noted that an earlier study by Farah et al. [8] showed no change in motor activity performed in adult mice undergoing fructose overload. Thus, further studies should be conducted to elucidate the mechanisms underlying this adaptation induced by fructose consumption during child development.

We observed an increase in the MPI parameter, which shows the global status of the heart, in SF group when compared to SC and TC groups, thus indicating a cardiac stress induced by fructose overload. In fact, our group has previously shown that fructose overload in adult male rats exhibited diastolic dysfunction and increased MPI [21], and exercise training performed during the same period eliminated all of these derangements [36]. A study conducted with patients with metabolic syndrome showed reversed E/A ratio, suggesting impaired LV relaxation, the first stage of LV diastolic dysfunction, and combined exercise training undertaken for a year did not improve LV diastolic function in those individuals [28, 44].

Concerning hemodynamic values, an increase in MAP and DAP in the SF group and resting bradycardia in the trained animals were observed, as expected. Resting bradycardia has been used as a marker of cardiovascular exercise training effectiveness. These findings may be associated with the improvement in cardiac autonomic modulation observed in TF animals when compared to SF animals. Additionally, we hypothesized that changes in cardiac pacemaker, as noted earlier in adult male rats [40] or old trained rats [17], or even changes in the autonomic control of heart rate (vagal and sympathetic tone), might be involved in the bradycardia observed in the TC group, since there were no differences between SC and TC groups in cardiac modulation in both time and frequency domain.

The increase in AP after chronic consumption of fructose in rodents has often been reported [8, 21, 36]. Giacchetti et al. [45] associated hypertension induced by fructose overload with increased expression of AT1a receptors in rats. Shinozaki et al. [46] demonstrated increased expression of angiotensin AT1 receptor and the angiotensin II pressor response in rats fed with a fructose-rich diet. Furthermore, there is evidence to support the role of a sympathetic nervous system fructose-induced hypertension. Verma et al. [47] showed that sympathectomy attenuated the elevation of AP in rats treated with fructose. Urinary excretion of catecholamine and adrenergic receptor expression were increased in animals treated with a fructose-rich diet [48]. Studies by our group observed increased AP, together with autonomic dysfunction, in male rats and mice treated with fructose, as well as in healthy females or hypertensive ovariectomized rats undergoing chronic consumption of fructose [6, 7, 8, 35, 37, 45].

These findings were associated with vascular and cardiac autonomic dysfunction. Indeed, in the present study we observed an increase in sympathetic-vagal balance (LF / HF) as well as in vascular sympathetic modulation, as demonstrated by the different *indexe*s evaluated by the variation in SAP; however, we did not find any difference in spontaneous baroreflex sensitivity in sedentary animals chronically treated with fructose. As such, our results point to an important sympathetic activation in cardiac and vascular tissues in animals treated with fructose, thus corroborating the other investigations above mentioned.

An important finding of this study was the normalization of MAP in the trained fructose group, which was accompanied by the normalization of the changes in the HR and SAP variability. In fact, this finding suggests that hemodynamic and autonomic dysfunctions in post-weaning rats treated with fructose and evaluated at a young age may be attenuated by moderate physical training when compared with those kept sedentary. These results are consistent with the findings previously published by our group for adult male rats [34, 39] and hypertensive ovariectomized female rats [35]. In this regard, it is notable that changes in the SAP variability have been related to lesions in target organs and worse prognosis [49].

In addition, please note that there is some evidence suggesting that the autonomic nervous system may be the triggering factor of the biomolecule secretion involved in the genesis of cardiometabolic disorders [50, 51]. Moreover, studies have reported that insulin resistance and hyperinsulinemia increased lipid peroxidation and decreased antioxidant systemically, suggesting that the two are interconnected [10, 11]. This hypothesis is supported by a recent study conducted by our group, in which we found that chronic consumption of fructose in male mice promoted increase in SAP and MAP and in cardiac and vascular sympathetic modulation along with attenuation of baroreflex from the 15th day of fructose consumption. However, it induced insulin resistance, increased plasma levels of cholesterol, triglycerides and leptin only after 60 days of fructose consumption. Such findings show that dysfunction of cardiovascular autonomic modulation occurred before any metabolic change [14].

In the present study lipid peroxidation (evaluated by CL) in LV was increased in the SF group, without any change in the protein damage profile. Such responses involved adaptations in antioxidant systems, such as: reduction of TRAP and SOD in LV of SF group when compared to the SC group. Moreover, systemically there was evidence of protein damage and lipid peroxidation in the SF group when compared to the SC group. In fact, Thirunavukkarasau and colleagues [52] showed an increase in systemic lipid peroxidation in mice consuming fructose. Furthermore, physical training reduced lipid peroxidation when compared to the sedentary group, and this improvement is associated with increased CAT and maintenance of the concentration of SOD in skeletal muscle [53]. Our group also found a reduction of membrane lipid peroxidation in old Wistar rats and hypertensive male rats undergoing physical training, as well as in female ovariectomized rats. Such changes were accompanied by an increase in enzymatic antioxidant defenses, and were correlated with improved cardiovascular and/or autonomic function [17, 54]. In this sense, in the present study we observed a reduction in LV and in plasmatic protein damage in TF group when compared to the SF group. Moreover, the TF group showed reduced lipoperoxidation evaluated by CL and TBARS when compared to SF group. Interesting, the TF group levels of lipoperoxidation (TBARS) and protein oxidation in LV was lower than TC rats, despite similar values of lipoperoxidation (evaluated by CL) was observed between TF and TC groups. These data suggest that training in fructose fed-rats during lifespan may induce additional adaptation in oxidative stress status than in control rats. Furthermore, exercise training induced ventricular increase in non-enzymatic antioxidants, as demonstrated by increased TRAP, and a normalization of SOD activity in TF group, as compared to just an increase in CAT activity in TC group. The higher antioxidant capacity in TF group is probably related with markedly improvement in glutathione redox balance on LV in TF group as compared to all other groups. Thus, we hypothesize that exercise training induced by improvement in redox balance in fructose fed-rats is associated with additional reduction in levels of lipid and protein damage, probably leading to prevention of cardiac and autonomic dysfunctions.

Although some limitations of the present study have to be addressed for instance, for example, the lack of corporal composition and insulin levels, the evaluation of citrate synthase in the muscle to better characterize the efficacy of training, as well as, further investigations on the mechanisms underlying preventive role of exercise training, our observations on the metabolic, cardiovascular, autonomic and oxidative stress profiles led us to conclude that exercise training proved to be an effective approach to prevent the derangements promoted by a high-fructose diet in rats after weaning.

## Conclusion

In conclusion, our findings indicates that cardiac, hemodynamic, autonomic and oxidative stress dysfunctions induced by fructose overload early in life in male rats may be prevented by moderate aerobic exercise training and may mitigate metabolic syndrome in adults.
